# Improving the paradigm of approaches to adolescent sexual and reproductive health

**DOI:** 10.1186/s12978-016-0191-3

**Published:** 2016-06-13

**Authors:** Kate F. Plourde, Suzanne Fischer, Joy Cunningham, Kristin Brady, Donna R. McCarraher

**Affiliations:** Research Utilization, GHPN—Global Health, Population, and Nutrition, FHI 360, 359 Blackwell Street, Suite 200, Durham, NC 27701 USA; Director, Youth Department, SED—Socio and Economic Development, FHI 360, 1825 Connecticut Avenue NW, Washington, DC 2009 USA; RMNCH—Reproductive, Maternal, Newborn, and Child Health, GHPN—Global Health, Population, and Nutrition, FHI 360, 359 Blackwell Street, Suite 200, Durham, NC 27701 USA

**Keywords:** Adolescent sexual and reproductive health, Positive youth development

## Abstract

Traditional approaches to improving adolescent sexual and reproductive health (ASRH) have focused on changing individual behavior, with little emphasis on addressing the factors that contribute to this behavior: biological changes; the influence of family and friends; the communities in which young people live; and access to economic and academic opportunities. This article provides an overview of the various factors that influence ASRH behaviors and outcomes and suggests an approach grounded in the principles of positive youth development to reduce risk factors and improve the protective factors that contribute to adolescents’ successful and healthy transition into adulthood.

## Background

Despite the great strides that have been made in the field of adolescent sexual and reproductive health (ASRH), many young people’s SRH needs remain largely unmet. Globally, rates of adolescent mortality now exceed those of early childhood mortality, with the exception of some very low income countries; complications related to childbirth are the second leading cause of death among adolescents ages 15–19; and despite steady declines in the global number of HIV-related deaths, among adolescents the number of HIV-related deaths has increased by 50 % [1–5]. Traditional approaches to improving ASRH outcomes have largely focused on changing individual behavior by raising awareness of the consequences of engaging in risky SRH behaviors [6, 7]. Over time, however, it became clear that understanding the consequences was not enough; young people had to gain the related skills and knowledge to *change* risky behaviors [8]. The field of ASRH has continued to evolve, and now it is understood that young people’s health behavior is largely influenced by a complex set of factors outside of their control: biological changes; the influence of family and friends; the communities in which young people live; and access to economic and academic opportunities [9, 10]. So, while it’s still important to provide young people with the knowledge and skills they need, it’s time we consider a new paradigm to enhance the SRH and overall well-being of adolescents. This paradigm must take into account the broader social and structural factors that influence health behaviors and be grounded in the principles of positive youth development.

## Factors that affect adolescent sexual and reproductive

### Biological changes

Adolescence is a time of transition that is marked by many physical, psychological, and social milestones. The biological changes that occur during this period affect young people’s SRH decision making and behavior [11]. Brain development and changes in hormone levels increase adolescents’ predisposition for risk-taking [11]. This propensity for risk-taking serves an important developmental purpose: it can prompt adolescents to explore social experiences and develop new skills [11, 12]. However, without support, this same predisposition can lead young people to engage in risky SRH behaviors [11, 12].

### Influence of family and friends

Strong social networks and connections are a critical component of a young person’s transition into adulthood [13, 14]. Through social networks and connections, young people gain access to opportunities, relationships, and support to further develop their identity, self-esteem, and sense of belonging. However, negative relationships with peers, partners, and family members are detrimental to ASRH. For example, peer norms can increase the likelihood of engaging in risky health behaviors, and partners might negatively influence SRH decision making, including the choice to have sex and whether to use a condom or other contraceptive method [8, 9]. In many countries, adolescent girls are likely to experience a higher degree of social isolation than boys are due to school drop-out, early marriage, and a range of other factors [13]. Social isolation increases the risk of sexual violence, HIV, and unplanned pregnancy [13, 15].

### The communities in which young people live

Young people’s SRH outcomes are often poorer in urban and disenfranchised neighborhoods [16–18]. Emerging evidence suggests that physical environments lacking adequate housing, health services, safe spaces, and sanitation are correlated with low self-efficacy and a higher incidence of pregnancy, HIV, and STIs among adolescents and youth [16–18]. Beyond the physical environment, the social environment at the community level also influences young people’s SRH. Higher levels of neighborhood violence and crime are associated with an increased risk of adolescent pregnancy and risky SRH behaviors [16]. Community norms, including those related to gender, influence young people’s ability to access SRH information and services and govern their SRH behavior and decisions [8, 19]. For example, cultural norms that value fertility contribute to low rates of contraceptive use and high rates of pregnancy among adolescents and youth, particularly among married youth [19, 20].

### Access to economic and academic opportunities

There is a known relationship between wealth inequality and negative SRH outcomes, including adolescent pregnancy [10]. Economic vulnerability can increase adolescents’ likelihood of engaging in intergenerational relationships, marrying early, and selling or trading sex, all of which increase their risk of HIV infection and early or unintended pregnancy [21].

### The new paradigm

Rather than focusing on factors that have negative impact, the field of positive youth development (PYD) seeks to build *protective* factors by intentionally engaging adults, communities, government agencies, schools, and young people themselves to provide opportunities for success [22, 23]. A PYD approach can be applied to address factors that affect SRH in the following ways.

- **Biological changes:** No approach can change biology, but we can help young people better cope with the biological changes typical of adolescence by providing age-appropriate and developmentally-appropriate sexuality education. Adolescents will gain information about safe behaviors and the skills needed to safely navigate the biological factors that may put them at risk [22, 23]. Research confirms that delivery of high-quality SRH information has a positive impact on young people’s attitudes and practice, including SRH-seeking behavior [24]. Sexual and reproductive health curricula that address gender and power are more likely to lead to reductions in sexually transmitted infections and decreases in unintended pregnancies among adolescents and youth [25].
- **Influence of family and friends**: Programs to strengthen family connections, increase association with positive peer groups, and provide safe spaces for young people to meet with peers all afford adolescents the opportunity to form meaningful relationships and contribute to positive SRH outcomes [13, 14]. For example, a randomized control trial examining the impact of an intervention to improve parent–adolescent communication about SRH found the intervention to be effective in improving young people’s knowledge about condoms and their self-efficacy to use them [26].
- **Communities in which young people live**: We can engage community members to transform norms and improve the physical surroundings in which young people live. Doing so helps create environments where adolescents feel safe and valued and that are supportive of their SRH [8]. For example, a program to delay first birth and improve birth spacing among married adolescents in India engaged community members in discussions about the health benefits of such practices. The program resulted in a significant increase in contraceptive use among married adolescents [27, 28].
- **Access to economic and academic opportunities**: Positive economic status and access to cash, credit, and savings can have a strong effect on SRH outcomes [25]. When combined with other social support and life skills, building adolescents’ financial capital can lead to reduced sexual risk-taking behavior, increased health knowledge, and increased service-seeking behavior [16, 25]. Additionally, higher rates of participation in education are associated with lower HIV prevalence among adolescents, fewer adolescent pregnancies, and delayed sexual initiation [17, 26, 27]. For example, beneficiaries of a program in Malawi that provides adolescents and their families cash transfers conditional on school attendance were more likely to stay in school, less likely to become pregnant, and more likely to avoid risky sexual and reproductive health behaviors [29].

## Conclusion

Adolescents need more than skills and information. To truly improve adolescents’ health outcomes we must also provide academic and economic opportunities, the space to develop positive adult and peer networks, and safe supportive environments. Thus, applying the principles of PYD requires coordination across developmental sectors. Replacing the problem-based approach with a comprehensive approach to positive youth development will challenge the organizational structures and funding mechanisms of many nongovernmental and government agencies. So it is critical that we become more creative, flexible, and integrated if we’re to effectively address the myriad issues that put young people at risk. If provided the proper support and environment to thrive, today’s generation of adolescents will become the world’s greatest asset.

## Abbreviations

ASRH, Adolescent Sexual and Reproductive Health; PYD, Positive Youth Development; SRH, Sexual and Reproductive Health
